# Metabolite Variations in the Hippocampus and Corpus Callosum of Patients with Mild Cognitive Impairment Using Magnetic Resonance Spectroscopy with Three-Dimensional Chemical Shift Images

**DOI:** 10.3390/brainsci13091244

**Published:** 2023-08-26

**Authors:** Yen-Lon Kau, I-Hung Lin, Chi-Long Juang, Chao-Kai Chang, Wen-Hsiang Ho, Hsiao-Chuan Wen

**Affiliations:** 1 Department of Medical Imaging, Camillian St. Mary’s Hospital, Luodong, Yilan 265502, Taiwan; gouyenlon@gmail.com (Y.-L.K.); smh02264@smh.org.tw (W.-H.H.); 2 Department of Medical Imaging and Radiological Sciences, Yuanpei University, Hsinchu 30015, Taiwan; cljuang@gm.ypu.edu.tw; 3 Nobel Eye Institute, Taipei 100008, Taiwan; petercard@gmail.com; 4 Department of Ophthalmology, Taipei Medical University Hospital, Taipei 11031, Taiwan; 5 Graduate Institute of Clinical Medicine, College of Medicine, National Taiwan University, Taipei 100, Taiwan; 6 Department of Optometry, Yuanpei University, Hsinchu 30015, Taiwan; chaokai@ms17.hinet.net; 7 Department of Pet Healthcare, Yuanpei University, Hsinchu 300, Taiwan

**Keywords:** mild cognitive impairment, magnetic resonance imaging, magnetic resonance spectroscopy, hippocampal gyrus, corpus callosum

## Abstract

This study compared the metabolites in the brain regions of hippocampus and corpus callosum between patients with mild cognitive impairment (MCI) and healthy controls using no-radiation and high-sensitivity magnetic resonance spectroscopy (MRS) with three-dimensional chemical shift images (3D-CSI). Twenty volunteers (seven patients with MCI and 13 healthy controls) aged 50–71 years were recruited for this prospective study. MRS with 3D-CSI images of a variety of metabolites was collected from the hippocampus and corpus callosum. Sex and weight showed no significant differences between the two groups. The metabolite levels in the hippocampus and corpus callosum of the MCI group were generally lower than in those of the healthy group, especially for creatine (*p* < 0.001 in the hippocampus and *p* = 0.020 in the corpus callosum) and N-acetyl aspartate/creatine (*p* < 0.001 in the hippocampus and *p* = 0.020 in the corpus callosum); however, choline/creatine showed a significant difference (*p* < 0.001) only in the hippocampus, and myo-inositol/creatine showed a significant difference (*p* < 0.001) only in the corpus callosum. Our study demonstrated that MRS with 3D-CSI can be used to measure these metabolite levels to determine the differences between patients with MCI and healthy individuals. This would aid early diagnosis of MCI in clinical practice, and patients could receive prompt intervention to improve their quality of life.

## 1. Introduction

Mild cognitive impairment (MCI) is common in the elderly, and is defined as a subjective and objective decline in memory, cognition, and function, greater than that in others of the same age and level of education, that does not meet the criteria for a diagnosis of dementia [1,2]. Patients with MCI have mild impairments in attention, memory, language, mental, and motor functions. Generally, their daily lives are unaffected, and only complex and detailed activities are disturbed. Previous research has shown that the memory and life function performance of patients with MCI is between those of normal elderly and patients with mild Alzheimer’s dementia [3]. It can be seen as a transitional stage between normal cognitive function and mild dementia, and approximately 12% of patients with MCI progress to mild dementia every year [4]. Although some patients with MCI may return to a normal cognitive function status, they are still in a high-risk group of degeneration into dementia. Therefore, MCI may be regarded as a very early stage of dementia. If MCI can be diagnosed early, it could be prevented from progressing to dementia through cognitive function training and controlling the risk factors related to progression to dementia.

Many subjective rating scales are used for diagnosing MCI, such as the Clinical Dementia Rating scale (CDR), Global Deterioration Scale (GDS), Mini-Mental State Examination (MMSE), and Alzheimer disease-8, (AD-8) [5,6]. However, unlike in dementia, changes in objective anatomical imaging due to mild cognitive impairment are difficult to observe. Patients with MCI may show a mildly decreased volume of the hippocampus or medial temporal lobe on structural magnetic resonance imaging (MRI) examination; however, the sensitivity and specificity of the test is low [7].

Another powerful objective tool for the early diagnosis of MCI is magnetic resonance spectroscopy (MRS). Compared with MRI, MRS is a relatively novel technique that provides a detailed picture of in vivo biochemistry. It classifies the magnetic resonance signals of metabolites into frequencies using the difference in the resonance frequency of the nuclei, depending on the structure and environment of the metabolites. Therefore, MRS can be used to identify information such as the type of metabolite and its concentration in human organs [8,9]. Furthermore, MRS can be combined with three-dimensional chemical-shift imaging (3D-CSI) techniques, in which metabolites are mapped throughout the voxel of interest, giving the spatial distribution of the metabolites [10]. Therefore, this combination enables metabolic and detailed information on the position of the human organ of focus [11,12], including the brain [13].

Compared with the low sensitivity and specificity of MRI, using high-sensitivity MRS with 3D-CSI to measure the signal intensity of the metabolites in the brain may potentially aid the early diagnosis of MCI, because the metabolites in the brain, such as choline (Cho), N-acetyl aspartate (NAA), creatine (Cr), and myo-inositol (MI), may show different signal intensities in patients with MCI when the neurons or myelin in the brain are damaged [9,14,15,16,17,18,19]. Brain areas for assessment depend on several factors. The hippocampus is crucial in memory and cognitive function, and is reportedly the first region to be damaged in dementia [20,21]. The corpus callosum, which integrates and transfers information from bilateral cerebral hemispheres to process sensory, motor, and high-level cognitive signals [19,22], was also noted with atrophy changes in patients with MCI [23,24]. Therefore, in our study, MRS with 3D-CSI was used to compare the metabolites in the brain regions of hippocampus and corpus callosum between patients with MCI and healthy controls, to identify changes in the metabolites in these regions as an indicator of early MCI.

## 2. Materials and Methods

### 2.1. Participants

This prospective study conformed to the guidelines of the Helsinki Declaration and was approved by the institutional review board of Camillians Saint Mary’s Hospital, Luodong (SMHIRB No: IRB106007; start date: 20 June 2017). From 1 February 2017 to 30 May 2017, a total of 20 participants (patients with MCI and healthy individuals) aged 50–71 were included. If patients had any other brain disease before, such as frontotemporal lobar degeneration, cerebral small vessel disease, or brain trauma, they were excluded. All participants completed the informed consent form, AD-8 assessment, and the patient basic information questionnaire. The basic information questionnaire included age, sex, and presence or absence of MCI. Patients were diagnosed with MCI based on the results of the AD-8 assessment. A score of 2–4 points was considered to indicate presence of MCI and 0–1 to be healthy; a score of 5–8 points indicated dementia [6], and such patients were excluded from the study. The 20 volunteers were divided into two groups based on MCI diagnosis: seven were in the MCI group (three men and four women; average age, 60 ± 3.7 years) and 13 in the healthy control group (four men and nine women; average age, 61.2 ± 5.7 years). In the MCI group, one of the subjects was excluded because of long-term drinking habits, migraine headaches, and sleeping pills.

### 2.2. Procedures

**Figure 1:**
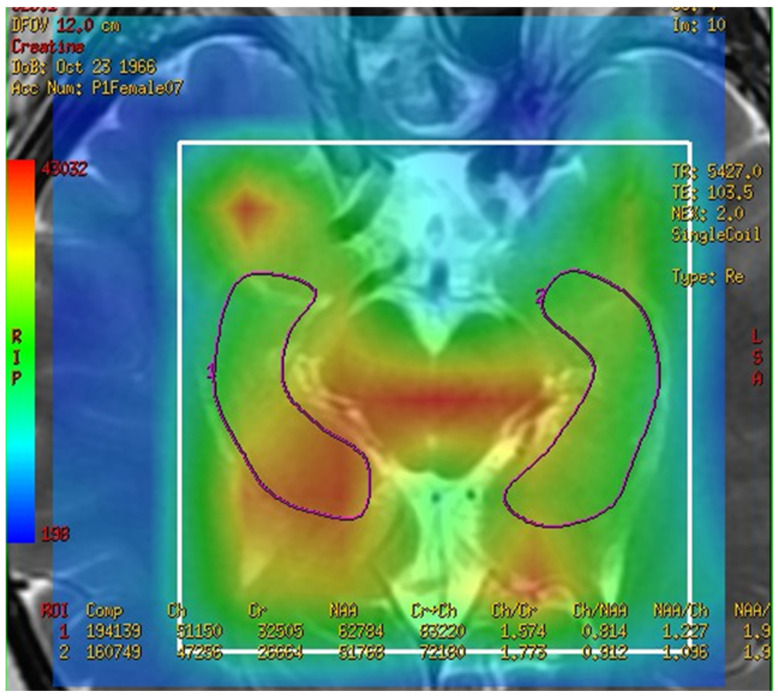
Magnetic resonance spectroscopy with three-dimensional chemical shift images in the chosen location of the left and right hippocampi (purple markup). The signal intensities of metabolites were measured and recorded.

**Figure 2:**
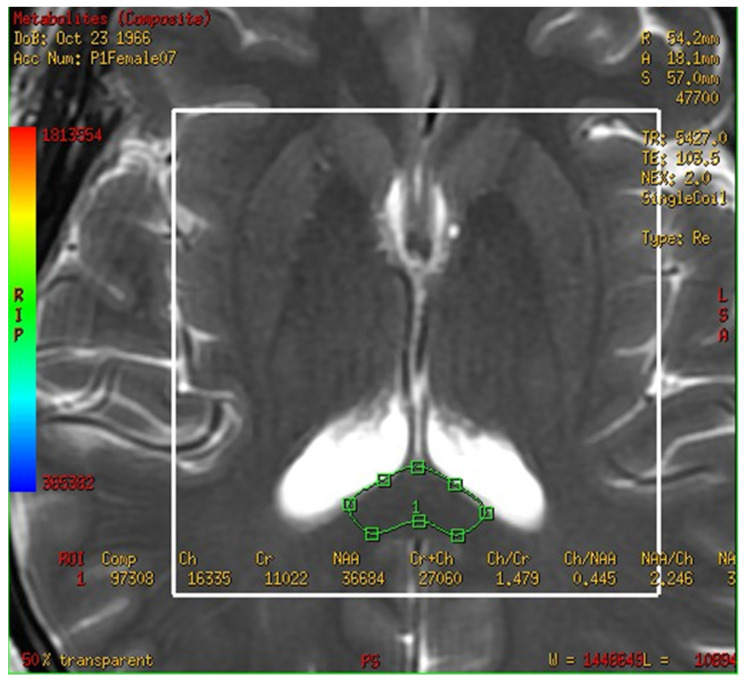
Magnetic resonance spectroscopy with three-dimensional chemical shift images in the chosen location of the bilateral corpus callosum (green markup). The signal intensities of metabolites were measured and recorded.

In this study, a medical 1.5 T MRI machine (GE Signa HDxt Medical Systems, Waukesha, WI, USA) and a standard head coil (Head Coil) in Camillians Saint Mary’s Hospital Luodong were used to receive signals from brain images of MRI T1 and T2 sequences, and ^1^H-MRS with 3D-CSI (echo time (TE) 144 ms, repetition time (TR) 1000 ms) for all participants. The range of chemical shift was set from 0 to 4 ppm, as this could include the signal of most metabolites in the brain, and the volume of each voxel was set at approximately 1–1.5 square centimeters. The measurements were performed as follows: First, brain images of T1 and T2 sequence with 3-Plane localization (sagittal, axial, and coronal planes) were obtained to locate the hippocampus and corpus callosum in the brain. Second, the three-dimensional space of the hippocampus and corpus callosum was manually marked and chosen on the image by a technician. Subsequently, the three-dimensional space of the hippocampus was divided into the total (bilateral), right, and left hippocampi. Third, the MRS with 3D-CSI was performed in the chosen location of the hippocampus (Figure 1) and corpus callosum (Figure 2). The signal intensities of choline (Cho), N-acetyl aspartate (NAA), creatine (Cr), and myo-inositol (MI) in the total (bilateral), right, and left hippocampi and the corpus callosum were measured and recorded in arbitrary units and used for statistical analysis.

### 2.3. Statistical Analysis

Statistical analyses were performed using the IBM SPSS for Windows software, version 20 (Armonk, NY, USA: IBM Corp). Due to the small sample size, the Mann–Whitney test was used to compare the signal intensities of metabolites between the MCI and control groups. Statistical significance was set at *p* < 0.05.

## 3. Results

### 3.1. Characteristics of the Participants

Table 1 shows the average age and weight of the participants. Mann–Whitney test of the two groups showed no significant difference in age and weight, indicating matched participants without selection bias. Moreover, sex showed no significant difference between the two groups (Table 1).

### 3.2. The Spectra of MRS Analysis in Brain and Fusion Map the 2-D Multi-Voxel Spectrum and Image

**Figure 3:**
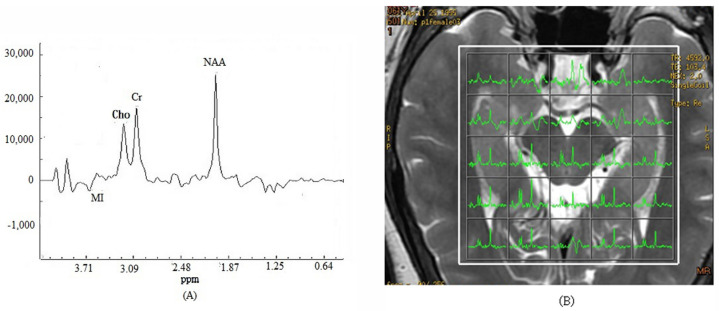
The multivoxel ^1^H Magnetic resonance spectroscopy with three-dimensional chemical shift images in one subject brain from 1.5 T MRI (**A**). The fusion map of 2-D multi-voxel spectrum and image from the same patient were analysis (**B**). *Y*-axis is the integral of peak.

The multivoxel ^1^H MRS of one subject from 1.5 T MRI is shown in Figure 3A. The fusion map of the 2-D multi-voxel spectrum and image from the same patient was shown in Figure 3B in one section.

### 3.3. Metabolites in the Total Hippocampus, Left Hippocampus, and Right Hippocampus in the MCI and Control Groups

The metabolites in the total hippocampus were compared between the MCI and control groups, and the ratio of normal Cr between two groups compared as follows: NAA/Cr (0.569 ± 0.341 vs. 1.714 ± 0.752, *p* = 0.001), Cho/Cr (0.410 ± 0.148 vs. 1.317 ± 0.614, *p* = 0.001), and the level of signal intensities of Cr (8900 ± 2400 vs. 29,000 ± 12,700, *p* = 0.001) were significantly lower in the MCI group (Table 2). The metabolites in the left hippocampus were compared between the MCI and control groups, and the ratio of normal Cr between two groups compared as follows: NAA/Cr (0.742 ± 0.621 vs. 1.663 ± 0.701, *p* = 0.030), Cho/Cr (0.443 ± 0.148 vs. 1.413 ± 0.644, *p* = 0.010), and the level of signal intensities of Cr (9500 ± 2800 vs. 26,400 ± 11,500, *p* = 0.010) were significantly lower in the MCI group (Table 2). Comparing the metabolites in the right hippocampus between the MCI and control group showed that the ratio of NAA/Cr (0.427 ± 0.212 vs. 1.759 ± 0.835, *p* = 0.001), Cho/Cr (0.383 ± 0.161 vs. 1.234 ± 0.604, *p* = 0.001), and the level of signal intensities of Cr (8300 ± 2900 vs. 31,600 ± 14,100, *p* = 0.001) were significantly lower in the MCI group (Table 2). For the levels of signal intensities of MI/Cr, there was no significant difference in the total hippocampus, left hippocampus, and right hippocampus compared between the MCI and control group.

### 3.4. Metabolites in the Corpus Callosum in the MCI and Control Groups

**Figure 4:**
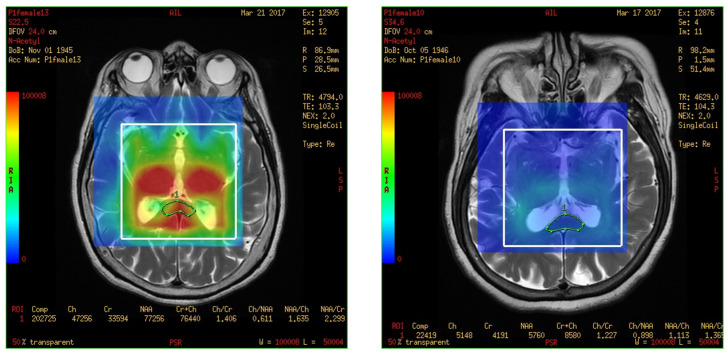
Magnetic resonance spectroscopy with three-dimensional chemical shift images color map of N-acetyl aspartate. The **left** figure shows red color (high signal intensity) in the corpus callosum area (green markup) of control group. The **right** figure shows blue color (low signal intensity) in the corpus callosum area (green markup) of mild cognitive impairment group.

On comparing the metabolites in the corpus callosum between the MCI and control group, the ratio of normal Cr between two groups compared as follows: NAA/Cr (0.778 ± 0.333 vs. 2.506 ± 1.648, *p* = 0.020), MI/Cr (0.062 ± 0.056 vs. 0.222 ± 0.136), and the level of signal intensities of Cr (4200 ± 2100 vs. 16,200 ± 11,800, *p* = 0.02) were significantly lower in the MCI group (Table 3). The result was easily visible in the color map of the MRS with 3D-CSI. For example, the MRS with 3D-CSI color map of NAA showed a red color (high signal) in the corpus callosum area of the control group, while it showed a blue color (low signal) in the corpus callosum area of the MCI group (Figure 4). For the levels of signal intensities of Cho/Cr, there was a trend of lower intensity at the corpus callosum in the MCI group compared with the control group, but without significant difference.

## 4. Discussion

This study found that, compared to healthy controls, the MCI group had significantly lower levels of signal intensities of NAA, Cr, and Cho, and had the same level of MI in the total, left, and right hippocampi. Further, the MCI group had significantly lower levels of signal intensities of NAA, Cr, and MI, and had a trend of lower intensity of Cho, but without significance in the corpus callosum, compared with healthy controls.

NAA, one of the most abundant biological metabolites in the brain, is a marker of mature neurons. It represents the functional state of brain cells and integrity of brain neurons [25]. Therefore, the reduction in NAA concentration may represent a reduction in the number of brain neurons. Diseases that damage the brain, such as tumors, hypoxia, abscesses, epilepsy, cranial neuropathies, and multiple sclerosis, can decrease its concentration [15,26]. Yang et al. found that patients with subjective cognitive decline plus showed significant decline in NAA in many brain areas, especially in the hippocampus and posterior cingulate cortex [27]. Gasparovic et al. found the NAA lever decrease as executive function decrease at the affected region of white matter of brain in patients of vascular cognitive impairment [28]. Kotb et al. found that patients with MCI and AD had lower hippocampal NAA level than patients with cognitive impairment associated with depression and healthy people [29]. Lidia et al. also found that the amount of NAA decreased in the whole brain of patients with MCI or Alzheimer’s disease [30]. Our study shows similar findings; additionally, we found that the decreased NAA in patients with MCI may be specifically localized in the hippocampus and corpus callosum.

Cho is an intermediate product of cell membrane phospholipid metabolism and is a marker of myelination, cell metabolism, and gliosis [17,18]. The Cho signal is mainly attributed to the presence of free glycerophosphocholine and phosphocholine when the cell membrane breaks down [31]. A decrease in Cho is reportedly indicative of decreased cell density, such as demyelination [32] or mitochondrial disturbance [33]. MCI is associated with demyelination [34] and neuronal cell death [35]. This may be attributable to why patients with MCI had a significantly decreased amount of Cho in the hippocampus, and also a decreasing amount of Cho in the corpus callosum in our study.

Cr, which serves as a reserve for high-energy phosphates in the cytosol of muscle and neurons and buffers cellular ATP/ADP reservoirs, is a marker of energy metabolism [18]. In patients with dementia or malignancy, cell necrosis occurs, and the energy metabolism pathways stop, decreasing the amount of Cr [9]. Oral Cr supplementation improved cognitive impairment in a rodent MCI model [36] and improved short-term memory and intelligence in healthy individuals [37]. Gasparovic et al. found the Cr lever decrease as executive function decrease in patients of vascular cognitive impairment [28]. Ulrich et al. found that the decline in Cr in the brain revealed by MRS can predict the conversion from MCI to dementia [38]. Catherine et al. found that the decline in Cr in the hippocampus revealed by MRS was noted in early Alzheimer’s disease and MCI [39]. These results are consistent with our finding and suggest that the decreased amount of Cr in the hippocampus and corpus callosum could be a biomarker for early diagnosis of MCI.

MI is involved in carbohydrate and lipid metabolism in the body. It usually exists in the form of phosphatidylinositol creatine. It is most abundant in the brain marrow, cardiac muscle, and skeletal muscle. It can regulate osmotic pressure, provide cell nutrition, and anti-aging effects [40]. However, its role in MCI remains unclear. Małgorzata et al. found that MCI was associated with higher MI concentrations in the parietal white matter than in healthy individuals [41]. However, Antonio et al. found no significant difference in the concentration of MI in the brain between MCI and healthy individuals. Our study showed that MCI was associated with a lower MI concentration in the corpus callosum, but the MI concentration level in the hippocampus was similar between MCI and healthy individuals. This may indicate that the change in MI concentration related to MCI was more sensitive in the corpus callosum than in the hippocampus.

## 5. Conclusions

Using MRS with 3D-CSI, the signal intensities of NAA, Cr, and Cho in the hippocampus and NAA, Cr, and MI in the corpus callosum was lower in patients with MCI than in healthy individuals. This may benefit early diagnosis of MCI in clinical practice, so that patients could receive prompt treatment and prevention of progression to dementia, and improve their quality of life. However, since the number of case and control group are small, further studies with larger sample sizes are needed.

## Figures and Tables

**Table 1:** Average age and weight of the participants.

	Control Group	MCI Group	*p* Value
Age (years)	61.3 ± 5.4	60.0 ± 3.7	0.476
Weight (Kilogram)	60.8 ± 9.1	66.4 ± 4.7	0.519

**Table 2:** Metabolites in the total hippocampus, left hippocampus, and right hippocampus compared between the MCI and control groups.

Metabolite	Control Group	MCI Group	*p* Value
Total hippocampus			
NAA/Cr	1.714 ± 0.752	0.569 ± 0.341	0.001 *
Cho/Cr	1.317 ± 0.614	0.410 ± 0.148	0.001 *
MI/Cr	0.313 ± 0.131	0.276 ± 0.262	0.475
Cr	29,000 ± 12,700	8900 ± 2400	0.001 *
Left hippocampus			
NAA/Cr	1.663 ± 0.701	0.742 ± 0.621	0.030 *
Cho/Cr	1.413 ± 0.644	0.443 ± 0.148	0.010 **
MI/Cr	0.333 ± 0.140	0.239 ± 0.186	0.320
Cr	26,400 ± 11,500	9500 ± 2800	0.010 **
Right hippocampus			
NAA/Cr	1.759 ± 0.835	0.427 ± 0.212	0.001 **
Cho/Cr	1.234 ± 0.604	0.383 ± 0.161	0.001 **
MI/Cr	0.297 ± 0.136	0.307 ± 0.041	0.320
Cr	31,600 ± 14,100	8300 ± 2900	0.001 **

* or ** denotes significant difference using the Mann–Whitney test; * *p* ≤ 0.05; ** *p* ≤ 0.01. The unit of signal intensity was arbitrary. Cho, choline; Cr, creatine; MI, myo-inositol; MCI, mild cognitive impairment; NAA, N-acetyl aspartate. Data was presented as mean ± standard deviation (SD).

**Table 3:** Metabolites in the corpus callosum compared between the MCI and control groups.

Metabolite	Control Group	MCI Group	*p* Value
NAA/Cr	2.506 ± 1.648	0.778 ± 0.333	0.020 *
Cho/Cr	1.327 ± 1.062	0.321 ± 0.204	0.113
MI/Cr	0.222 ± 0.136	0.062 ± 0.056	0.001 **
Cr	16,200 ± 11800	4200 ± 2100	0.020 *

* or ** denotes significant difference using the Mann–Whitney test; * *p* ≤ 0.05; ** *p* ≤ 0.01. Cho, choline; Cr, creatine; MI, myo-inositol; MCI, mild cognitive impairment; NAA, N-acetyl aspartate. Data was presented as mean ± standard deviation (SD).

## Data Availability

The datasets presented in this study are available on request from the corresponding author.
